# The FXR agonist obeticholic acid inhibits the cancerogenic potential of human cholangiocarcinoma

**DOI:** 10.1371/journal.pone.0210077

**Published:** 2019-01-24

**Authors:** S. Di Matteo, L. Nevi, D. Costantini, D. Overi, G. Carpino, S. Safarikia, F. Giulitti, C. Napoletano, E. Manzi, A. M. De Rose, F. Melandro, M. Bragazzi, P. B. Berloco, F. Giuliante, G. Grazi, A. Giorgi, V. Cardinale, L. Adorini, E. Gaudio, D. Alvaro

**Affiliations:** 1 Department of Translational and Precision Medicine, Sapienza University of Rome, Rome, Italy; 2 Department of Anatomical, Histological, Forensic Medicine and Orthopedics Sciences, Sapienza University of Rome, Rome, Italy; 3 Department of Movement, Human and Health Sciences, Division of Health Sciences, University of Rome "Foro Italico", Rome, Italy; 4 Department of Experimental Medicine, Sapienza University of Rome, Rome, Italy; 5 Gastroenterology Unit, Regina Elena National Cancer Institute, Rome, Italy; 6 Hepatobiliary Unit, Catholic University of the Sacred Heart School of Medicine, Rome, Italy; 7 Department of General Surgery and Organ Transplantation, Sapienza University of Rome, Rome, Italy; 8 Medical-Surgical and Biotechnologies Sciences, Polo Pontino, Sapienza University of Rome, Rome, Italy; 9 Intercept Pharmaceuticals, New York, New York, United States of America; Texas A&M University, UNITED STATES

## Abstract

Cholangiocarcinoma (CCA) is an aggressive cancer with high resistance to chemotherapeutics. CCA is enriched in cancer stem cells, which correlate with aggressiveness and prognosis. FXR, a member of the metabolic nuclear receptor family, is markedly down-regulated in human CCA. Our aim was to evaluate, in primary cultures of human intrahepatic CCA (iCCA), the effects of the FXR agonist obeticholic acid (OCA), a semisynthetic bile acid derivative, on their cancerogenic potential. Primary human iCCA cell cultures were prepared from surgical specimens of mucinous or mixed iCCA subtypes. Increasing concentrations (0–2.5 μM) of OCA were added to culture media and, after 3–10 days, effects on proliferation (MTS assay, cell population doubling time), apoptosis (annexin V-FITC/propidium iodide), cell migration and invasion (wound healing response and Matrigel invasion assay), and cancerogenic potential (spheroid formation, clonogenic assay, colony formation capacity) were evaluated. Results: FXR gene expression was downregulated (RT-qPCR) in iCCA cells vs normal human biliary tree stem cells (p < 0.05) and in mucinous iCCA vs mixed iCCA cells (p < 0.05) but was upregulated by addition of OCA. OCA significantly (p < 0.05) inhibited proliferation of both mucinous and mixed iCCA cells, starting at a concentration as low as 0.05 μM. Also, CDCA (but not UDCA) inhibited cell proliferation, although to a much lower extent than OCA, consistent with its different affinity for FXR. OCA significantly induced apoptosis of both iCCA subtypes and decreased their *in vitro* cancerogenic potential, as evaluated by impairment of colony and spheroid formation capacity and delayed wound healing and Matrigel invasion. In general, these effects were more evident in mixed than mucinous iCCA cells. When tested together with Gemcitabine and Cisplatin, OCA potentiated the anti-proliferative and pro-apoptotic effects of these chemotherapeutics, but mainly in mixed iCCA cells. OCA abolished the capacity of both mucinous and mixed iCCA cells to form colonies when administered together with Gemcitabine and Cisplatin. In subcutaneous xenografts of mixed iCCA cells, OCA alone or combined with Gemcitabine or Cisplatin markedly reduced the tumor size after 5 weeks of treatment by inducing necrosis of tumor mass and inhibiting cell proliferation. In conclusion, FXR is down-regulated in iCCA cells, and its activation by OCA results in anti-cancerogenic effects against mucinous and mixed iCCA cells, both *in vitro* and *in vivo*. The effects of OCA predominated in mixed iCCA cells, consistent with the lower aggressiveness and the higher FXR expression in this CCA subtype. These results, showing the FXR-mediated capacity of OCA to inhibit cholangiocarcinogenesis, represent the basis for testing OCA in clinical trials of CCA patients.

## Introduction

Intrahepatic cholangiocarcinoma (iCCA) is a cancer with both a poor prognosis and increasing incidence worldwide [1]. iCCA includes two different histological subtypes: the large bile duct type (mucinous) iCCAs, and the small bile duct type (mixed) iCCAs, where areas of hepatocytic differentiation and neoplastic ductular reaction are present within the tumor mass [2]. Unfortunately, only approximately 40% of iCCAs are suitable for surgical resection at diagnosis and only palliative therapy is possible in most of cases [3, 4]. Although new therapies are under investigation, the Cisplatin-Gemcitabine combination currently represents the standard of care with a survival gain of only 2–3 months [5]. Other than the typical features associated with chemoresistance, e.g. high expression of MDR proteins, which characterize this tumor, we have recently demonstrated that cancer stem cells (CSC) are highly represented in iCCAs in association with cells expressing epithelial/mesenchymal transition (EMT) traits [3]. These features have been associated with aggressiveness, chemo-resistance and a dismal prognosis of iCCA [3].

The farnesoid X receptor (FXR, NR1H4) is a member of the nuclear metabolic receptor superfamily which, among many functions, upon interaction with bile acids (BA) regulates their synthesis and enterohepatic circulation [6]. Consistent with this, FXR is highly expressed in cells involved in BA transport, including enterocytes, hepatocytes, and cholangiocytes [7–9]. In addition, FXR directly modulates more than 300 genes involved in lipid and glucose metabolism, inflammation, fibrosis, liver regeneration, cell differentiation and proliferation [8, 10, 11]. Indeed, the proliferation of hepatocytes and intestinal cells and the differentiation of stem cells into adult cells of the hepatocyte or intestinal lineages have been recently demonstrated to be directly regulated by FXR [8, 10, 11].

More recently, FXR has been investigated in relation with carcinogenesis. Specifically, in different cancers, FXR functions as a tumor suppressor gene [12]. In hepatocellular (HCC) and colon carcinomas, for example, a low FXR expression has been demonstrated and, consistently, FXR activation has resulted in a significant repression of liver and colon cancer progression, primarily mediated by FXR-dependent inhibition of β-catenin activity [13–15].

FXR is aberrantly down-regulated in both human CCA and CCA cell lines, where ectopic expression of miR-421 has been involved in downregulating FXR protein concentration and in promoting cell proliferation, colony formation and migration *in vitro* [16, 17]. Conversely, a decrease in miR-421 expression induced G_0_/G_1_ cell cycle arrest [16, 17]. These findings suggest that FXR activation could represent a novel therapeutic strategy for treatment of biliary tract cancer [16].

In this study, using primary cultures of human iCCA, we evaluated the expression of FXR and the effects *in vitro* and *in vivo* of the FXR agonist obeticholic acid (OCA, also known as INT-747), on the cancerogenic potential of human iCCA cells. OCA is a semi-synthetic bile acid derived from the endogenous primary human bile acid chenodeoxycholic acid (CDCA) and differs from CDCA by the addition of an ethyl group in the 6’ position which confers approximately 100-fold increased FXR agonism, relative to CDCA (the endogenous human FXR agonist) [18]. Our results indicate that OCA exerts *in vitro* and *in vivo* relevant anticancer effects against iCCA.

## Materials and methods

### iCCA primary cell cultures

Primary cell cultures were prepared, as previously described [19], from specimens of human iCCA obtained from patients submitted to surgical resection and classified as mucinous or mixed iCCA by PAS staining, according to Komuta M. *et al* [2], and morphological criteria. CCA cultures were maintained in H69 medium, a hormonally supplemented medium consisting in Dulbecco’s Modified Eagle Medium (DMEM) with high glucose/DMEM:F12 Nutrient mixture (1:1) (Gibco/BRL, Life Technologies srl., Milan, Italy) supplemented with 243 μg/ml of adenine (Sigma Aldrich, Milan, Italy), 5 μg/ml of insulin (Sigma Aldrich, Milan, Italy), 8 μg/ml of transferrin (Sigma Aldrich, Milan, Italy), 2.1 10^−3^ μg/ml of triiodothyronine (Sigma Aldrich, Milan, Italy), 6.2 ∙10^−1^ μg/ml hydrocortisone, 0.01μg/ml of human epidermal growth factor (hEGF) (Sigma Aldrich, Milan, Italy), 1 μg/ml of epinephrine (Sigma-Aldrich, Milan, Italy), 10% of fetal bovine serum (FBS, Gibco/BRL, Life Technologies, Milan, Italy), 60 μg/ml of penicillin (Gibco/BRL, Life Technologies srl, Milan, Italy), and 100 μg/ml of streptomycin (Gibco/BRL, Life Technologies srl, Milan, Italy). Primary cell cultures were maintained at 37°C in a humidified atmosphere of 5% CO_2_. The use of human materials was approved by our local Institutional Review Board and the research protocol was approved by the Ethics Committees of the Policlinico Umberto I, University Hospital. After appropriate discussion, patients indicated their consent to participate to the study by signing the appropriate informed consent.

In the present study, mucinous and mixed iCCA primary cell cultures were cultured for 40 passages. As controls, we used human biliary tree stem cells (hBTSCs) isolated, as previously described, from human biliary tree [20–22].

### OCA, Gemcitabine, Cisplatin

Obeticholic acid (OCA) was provided by Intercept Pharmaceuticals, Inc. San Diego, USA, and was prepared as stock solution in dimethyl sulfoxide (DMSO, CAS Number 67-68-5, Sigma-Aldrich, Milan, Italy) and then diluted (1:10^5^) in culture medium at the desired final concentration; the same amount of DMSO was added to controls. Stock solutions of OCA were freshly prepared every 15 days.

Gemcitabine hydrochloride (Sigma-Aldrich, CAS Number: 95058-81-4) was prepared as a stock solution in water and added in the culture media of iCCA cells after dilution (1:10^4^). Cisplatin (Sigma-Aldrich, CAS Number: 15663-27-1) was prepared as a stock solution in DMSO and added to cell cultures, after appropriate dilution (1: 10^5^) in H69 medium. We used the concentration of Gemcitabine (10 μM) and Cisplatin (20 μM) previously demonstrated to exert maximal inhibitory effects on cell proliferation in the same primary human iCCA cell cultures used in the present study [19].

Chenodeoxycholic acid (CDCA) and ursodeoxycholic acid (UDCA) were purchased from Sigma-Aldrich, diluted in DMSO and added into culture medium after dilution to reach the desired final concentration. The same amount of DMSO was added to controls.

During the experiments, the cell medium was refreshed every 3 days.

### RT-qPCR analyses of FXR, and ASBT in primary iCCA cultures

Total RNA was extracted from cell cultures by using TRIzol (Thermo Fisher Scientific inc., Waltham, USA) and 1-bromo-3-chloropropane 1:5. RNA obtained was purified by precipitation with isopropanol (2-propanol) and solubilized in solubilized in RNase-Free Water (GIBCO Thermo Fisher Scientific, Waltham, MA USA). Total mRNA extraction was quantified by Nanodrop (Thermo Fisher Scientific Inc., Waltham, USA).

The reverse transcription primed by 0.5μg Oligo(dT) 12–18 primers (Thermo Fisher Scientific Inc., Waltham, MA USA) was conducted with an amount of 300 ng of isolated RNA and with 200U of M-MLV reverse transcriptase (Invitrogen s.r.l.) according to manufacturer’s instructions.

Gene expression was determined by Real-Time PCR with a MX3000P instrument (Agilent, La Jolla, CA, USA) setting average cycle threshold (Ct) at 0.100 (dRn) from three replicas of each sample. PCR amplifications were conducted with 1 μl of cDNA template and SYBR Green PCR Master Mix (Thermofisher Scientific Inc.), 0.12 μM each of upstream and downstream primer for the gene analysed, and 0.5 μM of reference dye (ROX Thermofisher Scientific Inc.). All expression levels were normalized to the expression of GAPDH (housekeeping gene). Table 1 shows the details of primers used in the study.

**Table 1:** Sequences of primer pairs used for amplifying the genes of interest and the internal reference gene (GAPDH).

Gene_	*Primers* (5’-3’)
GAPDH	F- ‘AGCCACATCGCTCAGACAC’ R- ‘GCCCAATACGACCAAATCC’
FXR	F- ‘GATGCCTGTAACAAAGAAGCCCC’ R- ‘CACACAGTTGCCCCCGTTTTTAC’
ASBT	F- ‘TGTGTTGGCTTCCTCTGTCAG’ R- ‘GGCAGCATCCTATAATGAGCAC’
SHP	F- ‘TCAACCCCGATGTGCCAGG’ R- ‘GGTCGGAATGGACTTGAGGG’
Bcl-xL	F- ‘TGCAGGTATTGGTGAGTCGG-3’ R- ‘AAGCGTTCCTGGCCCTTTC’

Primers were designed by the PROBEFINDER software (https://www.roche-applied-science.com/sis/rtpcr/upl/index.jsp).

### Proliferation assay

Cell proliferation was evaluated by MTS assay (CellTiter 96 AQueous MTS Reagent Powder, Promega). Approximately of 3 x 10^3^ cells was seeded into 96-well plates in 100 μL of culture medium. After 24 hrs from seeding, the medium was replaced with fresh culture medium containing the desired concentrations of drugs. The MTS assay was performed after additional 72 hrs of exposure to drugs. MTS reagent was added in the medium according the manufacturer’s instructions. After 20 min. incubation at 37°C, in a humidified atmosphere at 5% CO2, absorbance at 492 nm was recorded with the absorbent microplate reader. Results were expressed as % changes with respect to controls considered equal to 100%.

### Population doubling time

Approximately 8 x 10^4^ cells / well were seeded in 6 multi-well plates. After 24 hrs the medium was replaced with fresh culture medium enriched with drugs or DMSO (control). Then the cells ware cultured for 72 hrs. The time to cell number duplication number (Population Doubling Time, PDT) was calculated as previously described [20].

### Apoptosis assay

Approximately 8 x 10^4^ cells / well were cultured in 6 multi-well plates in presence of DMSO (control) or drugs for 10 days. Thereafter, apoptosis was measured by staining with BD Pharmingen kit, including FITC Annexin V, to identify early apoptotic cells, used in conjunction with a vital dye propidium iodide (PI) to identify late apoptotic cells (i.e. FITC Annexin and PI positive). The Cells were analyzed by a BD FACS Canto Flow Cytometer (Becton, Dickinson and Company, NJ, USA). Ten thousand events were acquired and analyzed by BD FACSDiva software (Becton, Dickinson Company, NJ, USA). Results were expressed as percentage increase of apoptotic cells compared to untreated cells (controls).

### Colony forming capacity

Mixed and mucinous iCCA cells were seeded in 24 well plate (approx. 150 cells/well) and cultured in presence of DMSO (control) or drugs. After 10 days, colonies were fixed with 0.1% crystal violet in ethanol (Sigma-Aldrich), the excess dye was removed by washing and colonies were counted. A colony was considered a cluster of at least 50 cells. Then the crystal violet was dissolved, and the absorbance of the extracted dye was measured at 595 nm. Quantitative measurement of dimension of the colonies (Colony Dimension Index) in a single well was calculated as: = Absorbance 595nm / Number of colonies.

### Spheroid forming capacity

Formation of spheroids *in vitro* is considered a self-renewal index [23]. We demonstrated [3, 19] that iCCA cells form spheroids efficiently in non-attached conditions, reaching a size of 100–500 μm after 10 days in culture. As described Cardinale *et al*. 2015 [3], two thousand iCCA cells were cultured in a serum-free medium of DMEM with high glucose / DMEM:F12 mixture (1:1) (Gibco/BRL; Life Technologies) supplemented with 20 ng/mL EGF, 10 ng/mL FGF-2, and 1× B27 (Gibco/BRL; Life Technologies) into each well of 6-well Ultra-Low Attachment plates (Corning, Lowell, MA). After 10 days, spheroids were visualized, counted and sized using light microscopy. The results of tumor sphere formation assay were displayed as size changes of tumor spheres formed with or without treatment [23].

### Wound healing assay and Matrigel invasion assay

Wound healing assay was used for *in vitro* evaluation of cell migration. Using the tip of a sterile pipette with a diameter of 400 μm, a scratch was created on the confluent cell mono-layers in 12 well plate and the culture medium was replaced by fresh medium. The cells were monitored at 24 h intervals for 7 days and photographs collected at regular time intervals by a Nikon Camera. Cell migration was quantitatively evaluated by calculating the percentage (%) of the scratch area covered by cells at different time intervals with Photoshop (Adobe Inc., San Jose, CA USA) software.

The *in vitro* cell invasion assay was performed by using specific plates (Corning Matrigel Invasion Chamber 6-Well Plate 8.0 Micron). Approximately 90 x 10^3^ cells/well were plated in FBS-free H69 medium while, beneath these wells, H69 medium with FBS was added. After 24 h of cell culture, the test drugs were added to the wells. Cells present in the well and cultured in FBS free medium, migrated through the Matrigel to reach the lower portion, containing medium added with FBS. At the end of the experiment, H69 medium with and without FBS was removed by DPBS washings, cells were then blocked for 30 minutes using crystal violet staining (0.1% in ethanol). The migrated cells were then photographed by optical microscopy and images were analysed using ImageJ software. Cell migration was evaluated by computing the number of cells that migrated through the matrix and the results were expressed as a percentage of cells migrated compared to controls.

### iCCA xenografts (*in vivo* tumorigenicity)

Human subcutaneous xenografts of iCCA have been obtained as previously described [3]. Briefly, human mixed iCCA cells from primary cultures were injected (approximately 10^6^ cells in 100 μl Matrigel HyStem-C Cell Culture Scaffold, Sigma-Aldrich) subcutaneously in the right side of the male BALB/c nude mice, 4–5 weeks old (mean body weight 25 g, purchased from Charles River Laboratories, Wilmington, MA) maintained under standard conditions in a temperature-controlled environment (20–22 °C) under 12 h light–dark cycles, according to the institutional guidelines for animal care. Tumor xenograft formation was followed by macroscopic inspection. After 5 weeks, when the tumor size was approximately 100 mm^3^, mice were randomly divided into six groups (N = 5) and were treated with OCA, Cisplatin, Gemcitabine, OCA + Cisplatin, OCA + Gemcitabine or DPBS only (controls), for additional 5 weeks. Gemcitabine was prepared as a stock solution in water and administered, after dilution in DPBS (1: 10^4^), by tail intravenous injection (1.05 mg/Kg in 100 μl) 2 times per week. Cisplatin was prepared as a stock solution in DMSO and administered, after dilution in DPBS (1:10^5^), via intravenous injection (2.4 mg/Kg in 100 μl), 2 times per week. Mice received either a chow diet or a diet supplemented with OCA (0.03% w/w, 30 mg/kg/day). Tumor volume was calculated as described [24, 25] and expressed as the percentage of volume changes at the time t (V_t_) (i.e. after 5 weeks) in comparison with the basal volume at the time 0 (V_0_ = approx. 100 mm^3^ the formula of Percentage Increase (PI) was PI = [(Vt—V_0_)/V_0_]·100.

After 5 weeks, mice were sacrificed by cervical dislocation, tumors were then removed and fixed in formaldehyde solution for histology and immunohistochemical analysis (IHC), as described [3, 19]. Briefly, specimens were fixed in 10% buffered formalin, embedded in low-temperature–fusion paraffin and cut into 3- to 4-μm sections. Sections were stained with hematoxylin and eosin (H&E). Immunohistochemistry for Proliferating Cell Nuclear Antigen (PCNA) was performed. Briefly, endogenous peroxidase activity was blocked by 30-minute incubation in methanolic hydrogen peroxide (2.5%). Antigens were retrieved, as indicated by the vendor, by applying Proteinase K (Dako, code S3020; Glostrup, Denmark) for 10 min at room temperature. Sections were then incubated overnight at 4°C with primary antibody (PCNA, Dako, code M0879). Samples were then rinsed with phosphate-buffered saline and incubated with secondary biotinylated antibody and with Streptavidin-HRP (LSAB + System-HRP, Dako, code K0690). Diaminobenzidine (Dako, code K3468) was used as substrate, and sections were counterstained with hematoxylin. Sections were examined in a coded fashion by Leica Microsystems DM4500B Light Microscopy (Weltzlar, Germany), equipped with a Jenoptik Prog Res C10 Plus Videocam (Jena, Germany), and scanned by a digital scanner (Aperio Scanscope CS System, Aperio Digital Pathology, Leica Biosystems, Milan, Italy) and processed by ImageScope. Slides were evaluated independently by two researchers blind to the treatment group. The extent of necrotic area was evaluated on H&E slides using ImageScope software and expressed as percentage of necrotic area. The number of PCNA positive cells was automatically calculated by a specific algorithm on the entire section, and then expressed as percentage of PCNA positive cells.

### Statistical analysis

Data are presented as the arithmetic means ± standard deviation (SD) or standard error (SE), as indicated. Statistical analyses were conducted using the paired or unpaired Student’s t-test as appropriate and the analysis of the variance (ANOVA) when multiple comparisons were performed. p < 0.05 was considered statistically significant. For calculation of the maximal inhibitory concentration of OCA on cell proliferation, the Matlab software (The MathWorks, Inc.) was used.

## Results

### Characterization of primary human iCCA cell cultures

Primary human iCCA cell cultures, obtained from resected iCCA specimens have been extensively characterized for several markers at gene and protein levels, as previously described [3, 19, 26]. Specifically, our primary human iCCA cell cultures (i.e. mucinous and mixed iCCAs), after 20–40 passages, were particularly enriched in cells expressing cancer stem cell markers and EMT markers but were negative for markers of contaminant cells including hematopoietic cells (CD45), tumor-associated macrophages (CD163), activated hepatic stellate cells (GFAP), endothelial cells (CD31), fibroblast-activation protein (FAP), and stromal-derived factor (SDF1) [3, 19].

**Fig 1:**
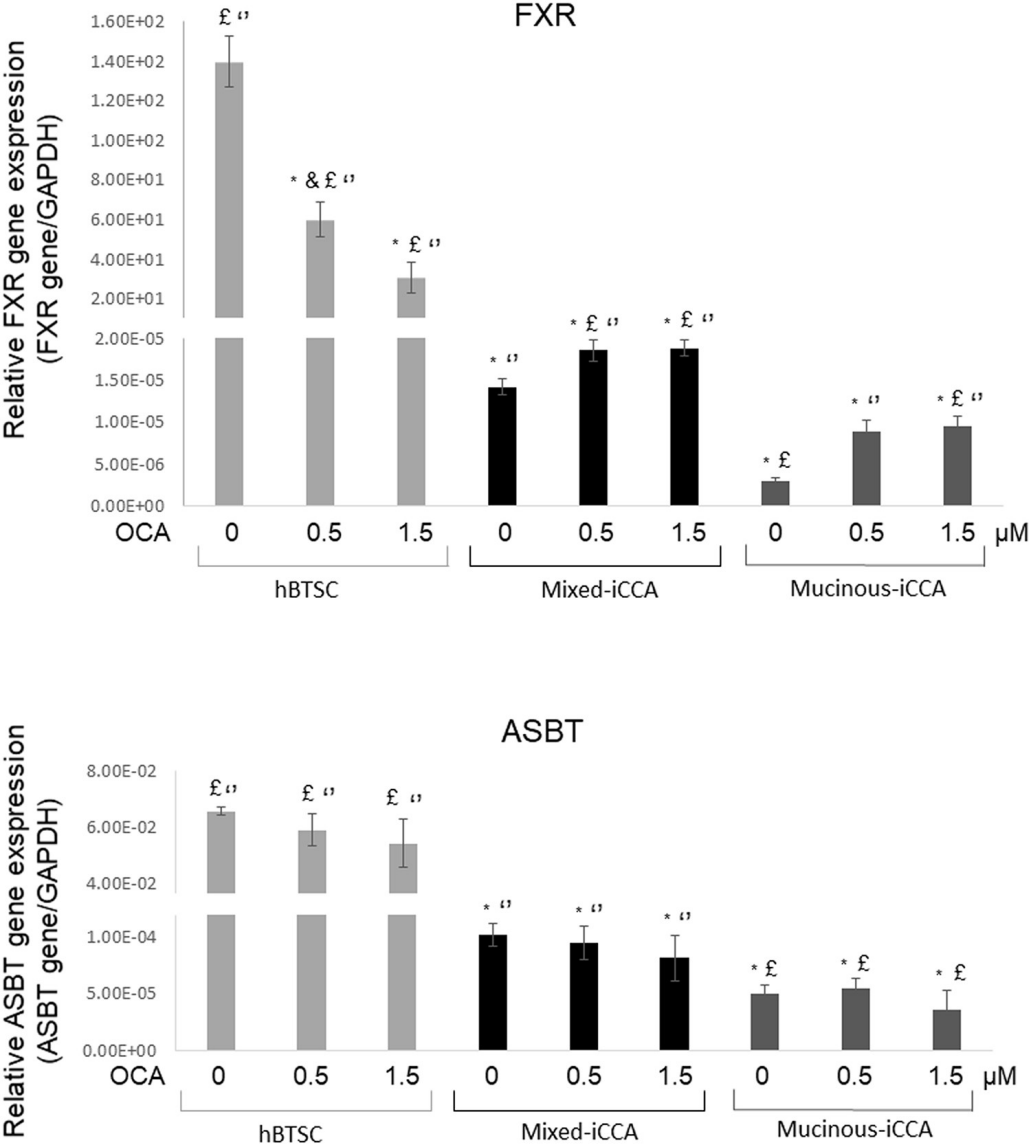
FXR and ASBT gene expression in mixed and mucinous iCCA primary cell cultures. The expression of FXR and ASBT in mixed and mucinous iCCA primary cell cultures was evaluated by RT-qPCR, using GAPDH as reference gene. Human biliary tree stem cells (hBTSCs) were used as control non-neoplastic cells given the high representation of cancer stem cell markers in CCA cells. The expression of FXR was markedly lower in mucinous iCCA and mixed iCCA cells compared to hBTSCs (p < 0.05). FXR gene expression increased after treatment with OCA 0.5 and 1.5 μM (p < 0.05). ASBT was similarly expressed in mucinous and mixed iCCA cells, but at a much lower level than in hBTSCs, and no significant difference was observed after treatment with OCA. Data represent means ± SD of N = 5 independent experiments. * p < 0.05 vs hBTSC control without OCA (0 μM OCA); ^£^ p < 0.05 vs mixed iCCA control without OCA (0 μM OCA); ‘‘ p < 0.05 vs mucinous iCCA control without OCA (0 μM OCA).

FXR expression was markedly lower (p < 0.01) in mixed and mucinous iCCA cells (without OCA) compared to human biliary tree stem cells (hBTSCs, without OCA) used as non-neoplastic controls (Fig 1). Cells obtained from mucinous iCCAs showed a lower expression of FXR compared to mixed iCCAs (p < 0.05). When treated with 0.5 μM and 1.5 μM OCA for 72 hrs, both mixed and mucinous iCCA primary cells increased the expression of FXR compared to untreated cells (p < 0.05), although not in a dose dependent manner (p> 0.05 0.5μM vs 1.5μM OCA). Conversely, FXR gene expression was decreased in control cells treated with 0.5 μM and 1.5 μM OCA for 72 hrs (p < 0.05).

ASBT, the sodium-dependent bile salt transporter, was also expressed in iCCA cells, but at a lower level than hBTSCs (without OCA). OCA treatment (0.5μM and 1.5μM for 72 hrs) had no effect on ASBT gene expression (p> 0.05) (Fig 1).

### OCA inhibits iCCA cell proliferation

**Fig 2:**
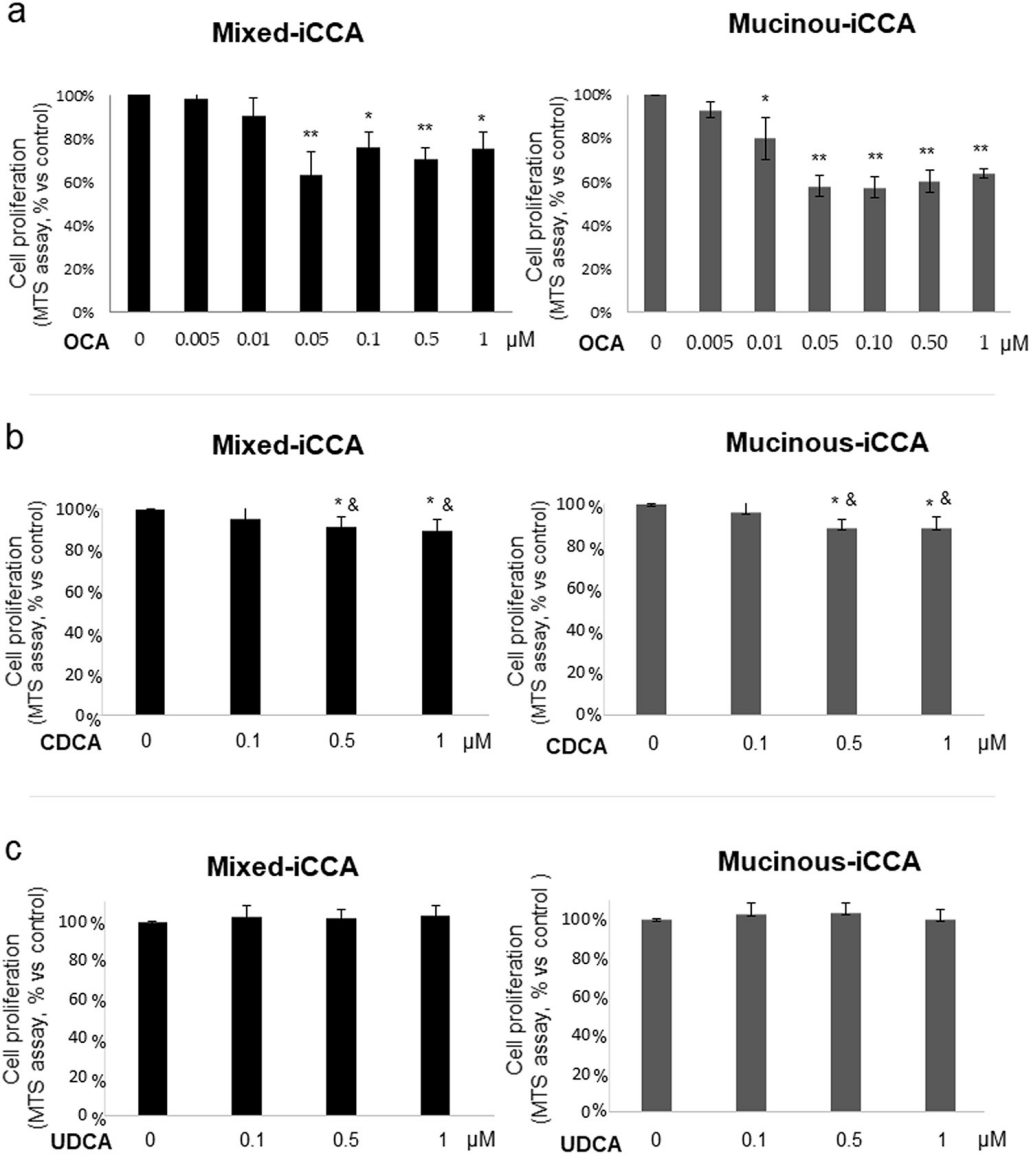
Effect of OCA, CDCA and UDCA on proliferation of primary human mixed and mucinous iCCA cell cultures. Cell proliferation was evaluated by MTS assay and expressed as percentage value with respect to controls (= 100%). In mixed iCCA, the inhibitory effect of OCA (72 hrs of incubation) on cell proliferation started to be significant at 0.05 μM concentration. In mucinous iCCA, the inhibitory effects of OCA on cell proliferation started to be significant at 0.01 μM. After a 72 hrs treatment, CDCA impaired cell proliferation of both mixed and mucinous iCCA primary cell cultures only at concentrations higher than 0.5 μM, but at a lower level than OCA. UDCA failed to influence cell proliferation in both mixed and mucinous iCCA primary cultures after 72 hrs of treatment. Data represent means ± SD of N = 5 independent experiments. * p < 0.05 vs controls without OCA (0 μM OCA); ** p < 0.01 vs controls; ^&^ p < 0.05 vs the same concentration of OCA.

In mixed iCCA cells (Fig 2a), the inhibitory effect of OCA (72 hrs of incubation) on cell proliferation was significant at a concentration of 0.05 μM and above. Maximum (38%) inhibition was calculated by Matlab software as 0.38 μM. In mucinous iCCA cells (Fig 2a), the inhibitory effects of OCA on cell proliferation was significant at 0.01 μM and higher (maximal inhibition of proliferation = 42% at 0.5 μM). The inhibitory effect on cell proliferation (in both mucinous or mixed iCCA cells) was not further enhanced by increasing the concentration of OCA up to 2.5 μM (not shown, N = 3 experiments each at 1.5, 2 and 2.5 μM OCA).

CDCA, an endogenous natural agonist of FXR, reduced cell proliferation (Fig 2b) of both mixed and mucinous iCCA primary cell cultures after a 72 hrs treatment. However, CDCA starts to exert inhibitory effects on iCCA cell proliferation only at a concentration higher than 0.5 μM (vs 0.05 μM of OCA), and the maximum inhibitory effect (approx. 10%) was significantly lower (p < 0.01) compared to OCA (approx. 35%). CDCA concentration up to 2.5 μM did not enhance the inhibitory effect on cell proliferation (not shown).

UDCA, which does not activate FXR, failed to influence cell proliferation of both mixed and mucinous iCCA primary cultures after a 72 hrs treatment, at concentrations of 0.1–1 μM (Fig 2c). Higher concentration of UDCA up to 2.5 μM had no effect on cell proliferation (not shown).

In primary cultures of iCCA cells exposed to OCA, we calculated the duplication time as an estimate of their proliferation rates. In mixed and mucinous iCCA cells, OCA increased the cell population doubling time (PDT). Indeed, in mixed iCCA cells, PDT was 0.58 ± 0.03 days in standard culture conditions (control), 0.83 ± 0.05 days with 0.5 μM OCA, 1.12 ± 0.05 days with 1 μM of OCA, 1.46 ± 0.06 days with 1.5 μM OCA (p < 0.05). In mucinous iCCA cells, PDT was 0.64 ± 0.03 days in control cells, 0.92 ± 0.04 with 0.5 μM OCA, 1.02 ± 0.03 with 1 μM OCA and 1.34 ± 0.05 with 1.5 μM OCA (p < 0.05).

### OCA enhances apoptosis in mixed iCCA cells

To examine if inhibition of iCCA cell viability was attributable to an increase in apoptosis, Annexin V-FITC/PI double labeling flow cytometry was carried out.

**Fig 3:**
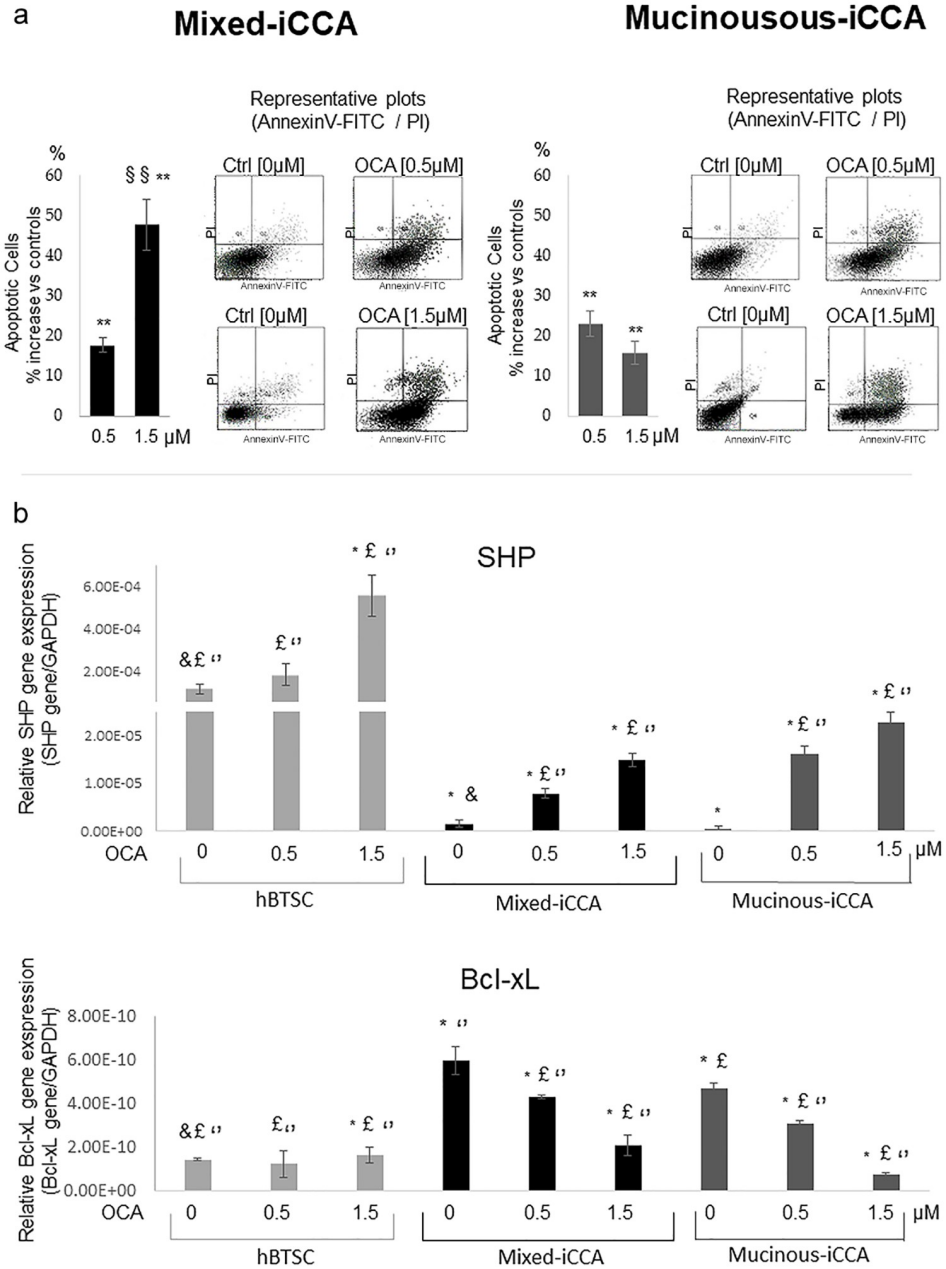
OCA enhances apoptosis in mixed and less in mucinous iCCA cells. a) Apoptosis was analysed by the Annexin V/PI assay in both mixed and mucinous iCCA cell cultures incubated for 72 hrs with 0.5 or 1.5 μM OCA. Results are expressed as percent increase of apoptotic cells with respect to controls. OCA increases apoptosis in both mixed and mucinous iCCA cell cultures. However, the apoptotic effect of OCA on mixed iCCA cells is more evident at 1.5 μM than 0.5 μM while no significant difference could be observed in mucinous iCCA cells between the two concentrations of OCA tested (0.5 and 1.5 μM). Data represent means ± SD of N = 5. Independent experiments. ** p < 0.01 vs controls without OCA (0 μM OCA); ^§§^ p < 0.01 vs 0.5 μM. b) OCA increases the expression levels of SHP gene supporting the activation of OCA pathways and the down-regulation of Bcl-xL gene, confirming the activation of apoptotic pathways. Data represent means ± SD of N = 5 independent experiments. * p < 0.05 vs hBTSC control without OCA (0 μM OCA); ^£^ p < 0.05 vs mixed iCCA control without OCA (0 μM OCA); ‘‘ p < 0.05 vs mucinous iCCA control without OCA (0 μM OCA).

OCA, after 72 hrs of treatment, increased apoptosis in both mixed and mucinous iCCA cell cultures, as demonstrated by the Annexin V/PI assay. However, the apoptotic effect of OCA on mixed iCCA cells was more evident at 1.5 than 0.5 μM (p < 0.05), while the percentage of apoptotic cells did not differ between 0.5 and 1.5 μM OCA in mucinous iCCA cells (Fig 3).

SHP gene expression in mixed and mucinous iCCA primary cell culture increased after 72 hrs of treatment with OCA 0.5 μM and 1.5 μM in a dose dependent manner (p < 0.05 vs 0μM; p < 0.05 0.5μM vs 1.5 μM). The control cells hBTSCs also increased the SHP gene expression after 72 hrs of treatment with OCA 0.5 μM and 1.5 μM.

Bcl-xL gene expression decreased after treatment for 72 hrs with OCA 0.5 and 1.5 μM in mixed and mucinous iCCA primary cell cultures (p < 0.05). No effect of OCA 0.5 μM and 1.5 μM (72 hrs of treatment) on hBTCS cells was observed.

### OCA inhibits the colony formation capacity of iCCA cells

**Fig 4:**
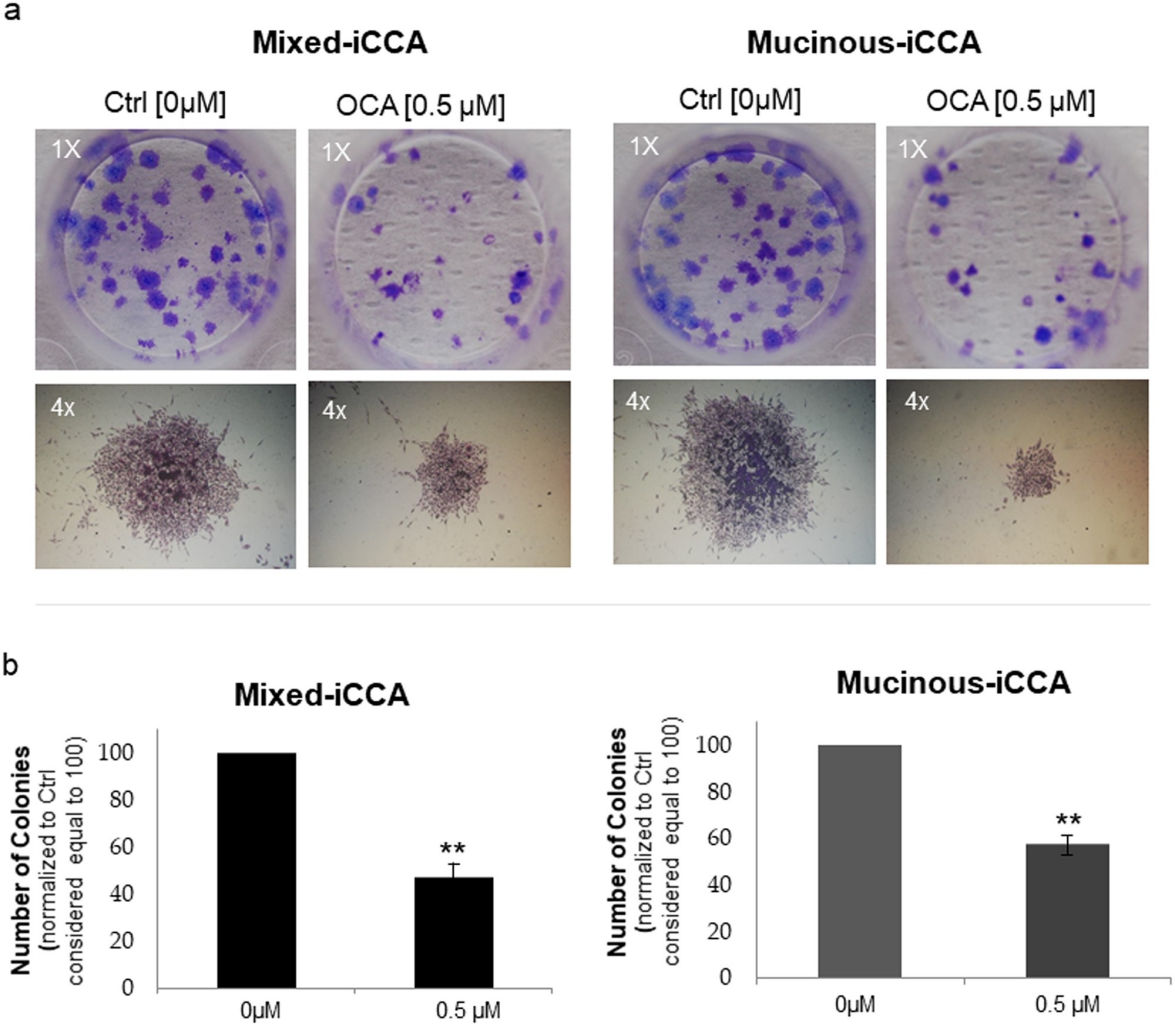
OCA inhibits the colony formation capacity of iCCA cells. After 10 days of culture, mixed and mucinous iCCA colonies were stained with 0.1% crystal violet and counted (a colony was considered a cluster of at least 50 cells). a) Photographs (magnification 1x) show representative wells in which mixed and mucinous iCCA colonies appear as a violet dot. The number of colonies decreases in the presence of 0.5 μM OCA. Photographs (magnification 4x) show representative colonies formed by mixed and mucinous iCCA cells, with evident decreased dimension induced by 0.5 μM OCA. b) The number of colonies was normalised to control (0 μM OCA) considered equal to 100. The number of colonies formed by both mucinous and mixed iCCA were significantly decreased by 0.5 μM OCA with respect to untreated cells. Data represent means ± SD of N = 5 independent experiments. ** p < 0.01 vs controls without OCA (0 μM OCA).

As shown in Fig 4, the number of colonies formed by both mucinous and mixed iCCA cells was significantly decreased (p < 0.05) by treatment with 0.5 μM OCA for 10 days compared to untreated cells (controls). The average colony size was also significantly decreased by treatment of both mixed and mucinous iCCA cells with 0.5 μM OCA. Indeed, the colony dimension index of mixed iCCA cells treated with OCA (3.34 ± 0.38, p < 0.05 vs controls) was significantly lower compared to untreated controls (4.15 ± 0.32, p < 0.05), and similar findings were found for mucinous iCCA cells (colony dimension index of OCA-treated cells = 2.16 ± 0.55 vs 4.56 ± 0.30 in untreated controls, p < 0.05).

### OCA inhibits spheroid formation in iCCA cells

**Fig 5:**
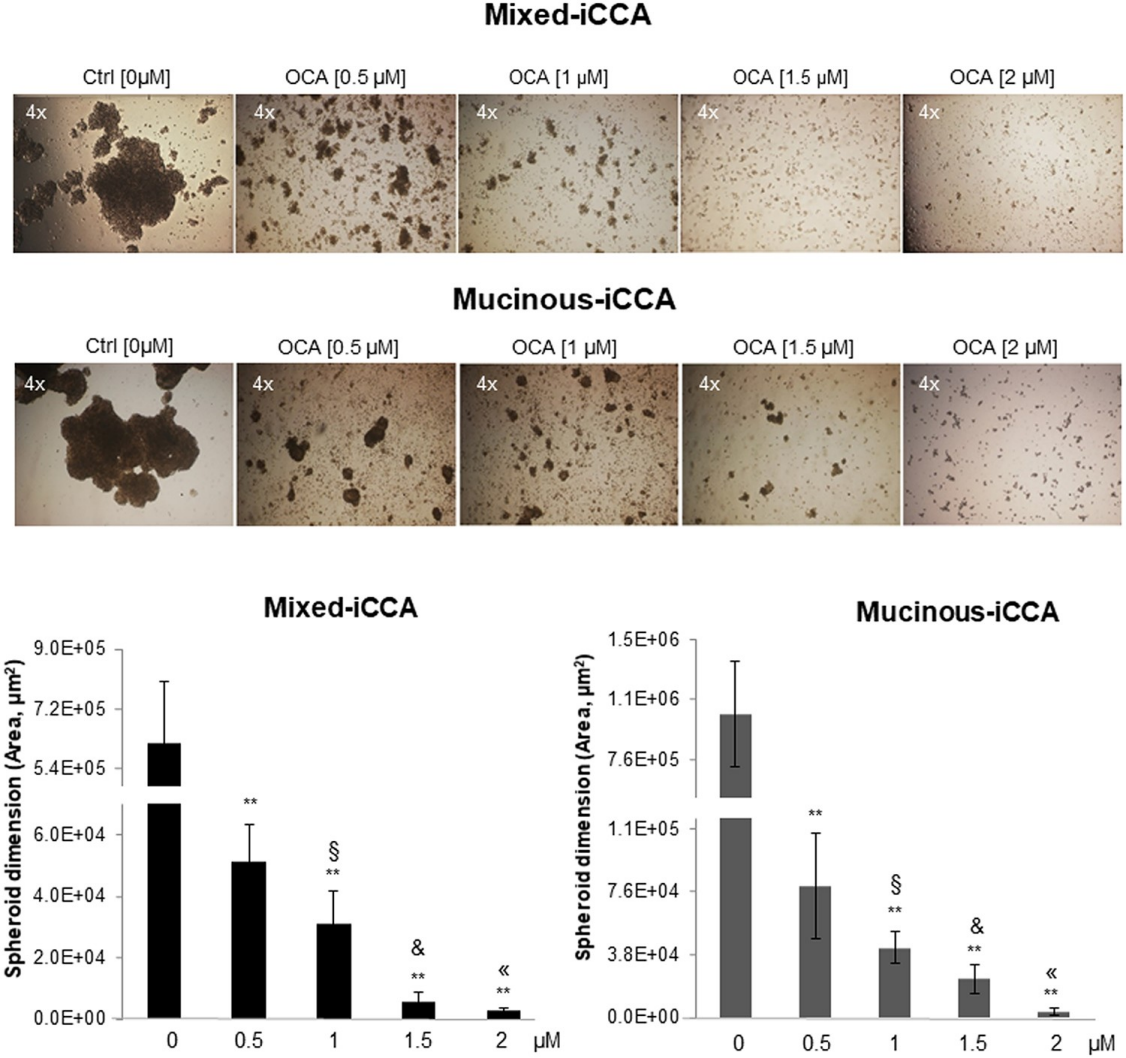
OCA inhibits spheroid formation in iCCA cells. Spheroid formation in non-adhesion conditions was compared between OCA treated and untreated control cells. OCA treatment (10 days) impaired formation of spheroids by both mixed and mucinous iCCA cells, and these effects were dose-dependent. At the highest concentration of 2 μM, spheroid formation was almost totally impaired by OCA. Data represent means ± SD of N = 15 independent experiments. ** p < 0.01 vs controls without OCA (0 μM OCA); ^§^ p < 0.05 vs 0.5 μM OCA; & p < 0.05 vs 1 μM OCA; « p < 0.05 vs 1.5 μM OCA.

Formation of spheroids by both mixed and mucinous iCCA cells was markedly impaired by a 10-day treatment with OCA compared to untreated cells, and these effects were dose-dependent (Fig 5). Specifically, spheroid formation was almost totally impaired by OCA at the highest concentration tested (2 μM).

### OCA impairs cell migration of mixed iCCA cells

**Fig 6:**
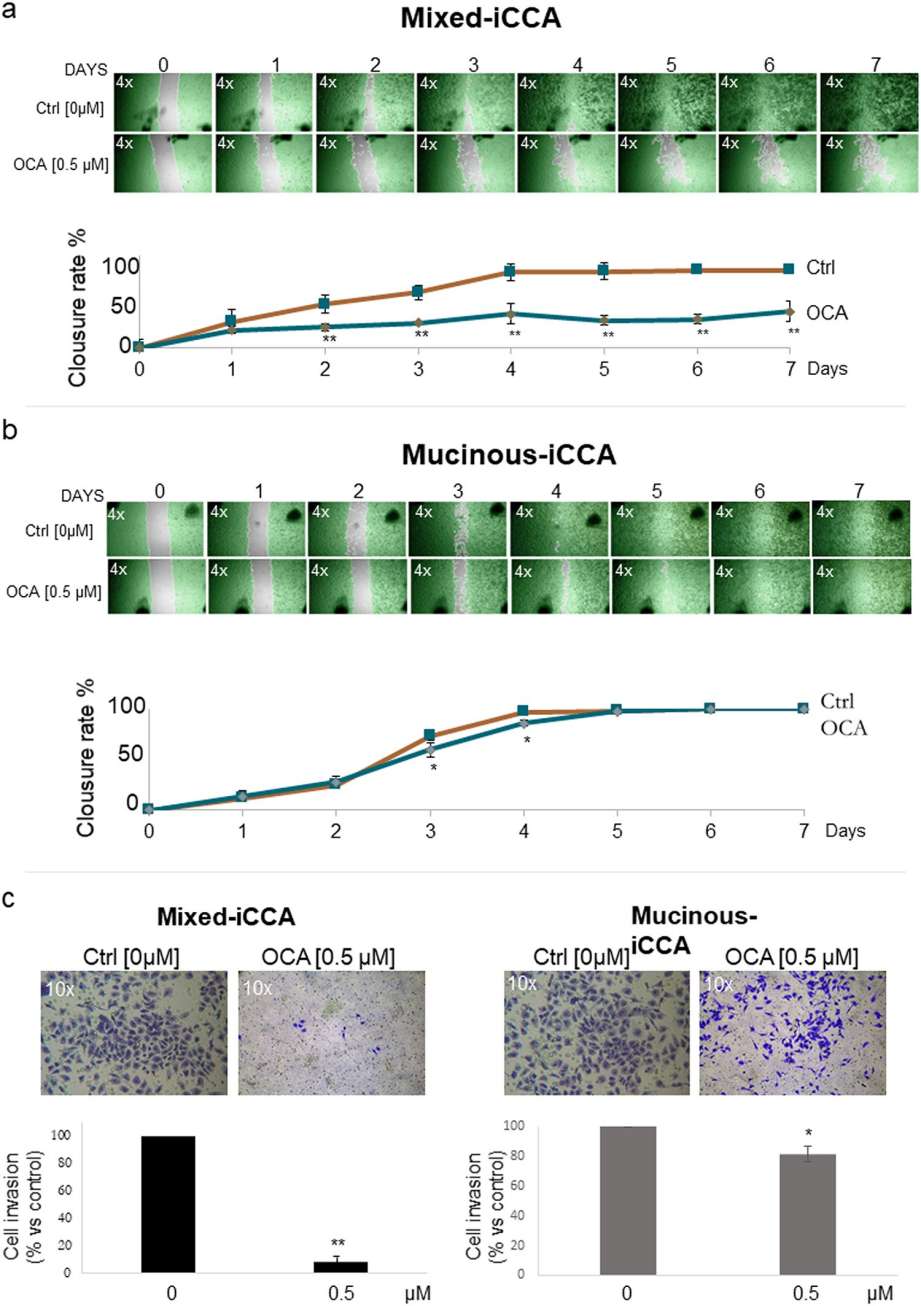
OCA impairs cell migration of mixed iCCA cells. a) Wound healing assay of mixed iCCA. A scratch was created on the confluent cell mono-layers and cells were monitored at 24 h intervals for 7 days. Cell migration was evaluated by calculating the percentage of covered area (% closure rate) at different time points vs time 0. Mixed control iCCA cells started to cover scratches after 2 days, and the wound healing was completed after 10 days. In contrast, in OCA (0.5 μM) treated cells, the scratches remained virtually uncovered until 7 days, indicating that migration of Mixed iCCA cells was almost completely impaired by OCA. Photographs (4x magnification) are representative of N = 6 independent experiments in which cells are highlighted in green and the cell-free area in grey. Data represent means ± SD of N = 6 independent experiments. ** p < 0.01 vs controls without OCA (0 μM OCA). b) In mucinous iCCA cells, in contrast, OCA showed no significant effect on cell migration; a slight inhibitory effect was seen only at days 3 and 4. Photographs (4x magnification) are representative of N = 6 independent experiments. Data represent means ± SD. * p < 0.05 vs controls without OCA (0 μM OCA). c) Cell migration was also evaluated by the Matrigel invasion assay, where the ability of tumor cells to migrate through the synthetic extracellular matrix was measured. After 96 h of culture, cells migrated through Matrigel were fixed with 0.1% crystal violet and their number reported as percentage respect to controls considered equal to 100 (see bar graph). OCA (0.5 μM for 4 days) impaired the ability to invade the Matrigel mainly in mixed iCCA and with minor effects in mucinous iCCA cells. 10x photographs were representative of N = 6 independent experiments. Means ± SD of N 6 independent experiments. ** p < 0.01 vs controls without OCA (0 μM OCA); * p < 0.05 vs controls without OCA (0 μM OCA).

As evaluated by the wound healing assay, OCA impaired cell migration of mixed iCCA cell cultures compared to untreated cells (Fig 6a). Mixed iCCA control cells started to cover scratches after 2 days and the wound healing was completed after 7 days. In contrast, in 0.5 μM OCA treated cells, the scratches remained virtually uncovered until 7 days, indicating that migration of mixed iCCA cells was almost completely impaired by OCA.

In mucinous iCCA, OCA showed no significant effect on cell migration. Indeed, untreated and treated cells showed a similar capacity to cover the scratches, indicating a comparable wound healing response. A slight but significant inhibitory effect was only seen at days 3 and 4 of culture (Fig 6b).

Cell migration was also evaluated by the Matrigel invasion assay, where the ability of the tumor cells to migrate through the synthetic extracellular matrix was measured after 96 hrs. Fig 6c shows how OCA (0.5 μM) impaired the ability of both mixed and mucinous iCCA cells to invade the Matrigel. Specifically, OCA decreased the Matrigel invasion ability of mixed iCCA cells by 91.50% (p < 0.01) and by 18.65% in mucinous iCCA cells (p < 0.05), compared to controls, after 4 days of treatment.

### OCA potentiates the inhibitory effects of Gemcitabine or Cisplatin on iCCA cell proliferation and apoptosis

**Fig 7:**
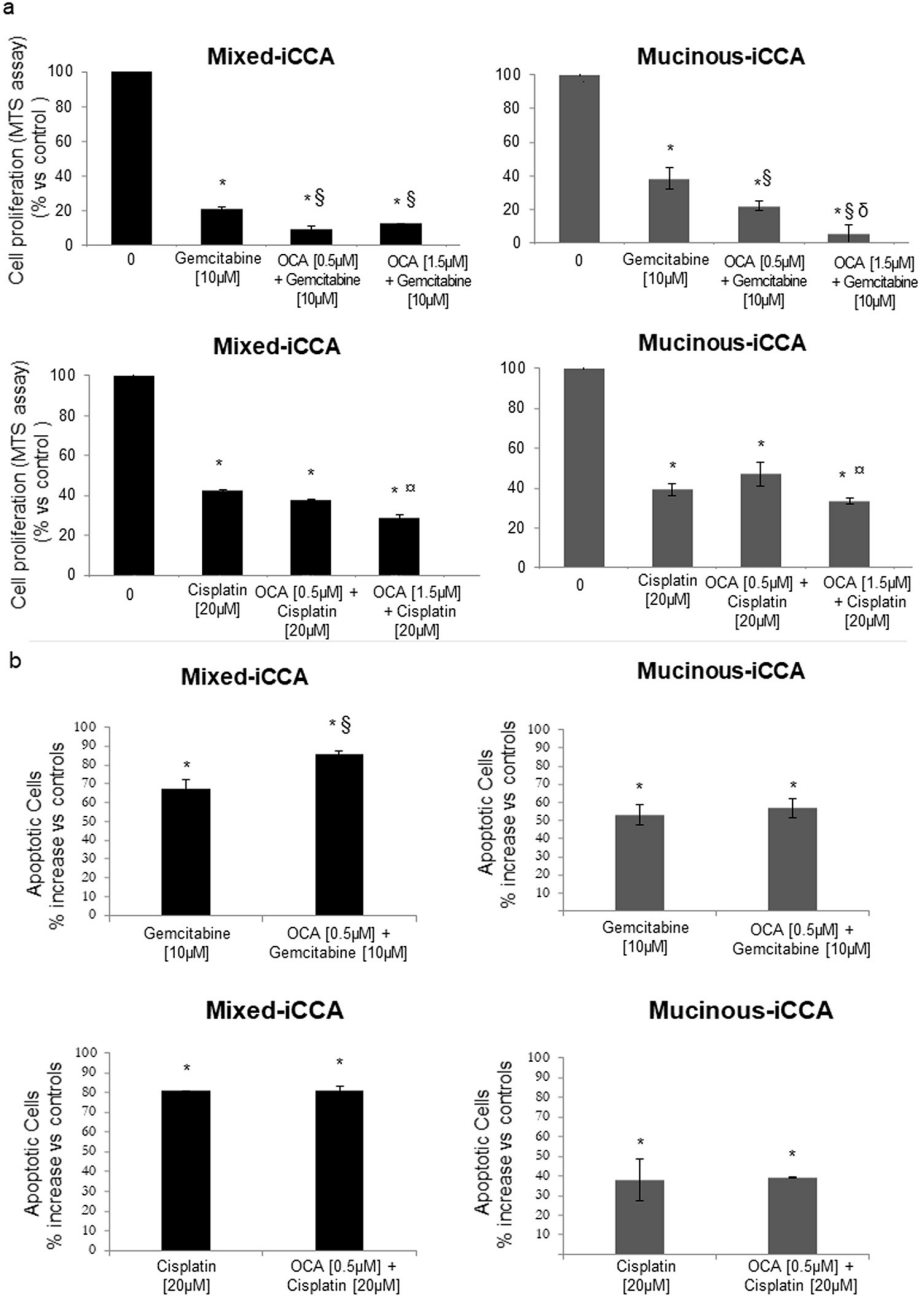
OCA potentiates the inhibitory effects of Gemcitabine or Cisplatin on iCCA cell proliferation and apoptosis. a) Cell proliferation was evaluated by MTS assay, after 72 hrs of treatment with OCA, ± Gemcitabine or Cisplatin, and was expressed as percentage of untreated controls (= 100). In mixed and mucinous iCCA, OCA potentiated the inhibitory effect of Gemcitabine on cell proliferation. While in mixed iCCA cells no difference was found between low (0.5 μM) and high (1.5 μM) OCA concentrations, in mucinous iCCA, in contrast, 1.5 μM OCA was more effective than 0.5 μM in potentiating the effects of Gemcitabine (p < 0.05). The inhibitory effect of Cisplatin on proliferation of both mixed and mucinous iCCA cells was potentiated by OCA but only at the highest concentration tested (1.5 μM). Data represent means ± SD of N = 12 independent experiments. * p < 0.05 vs controls without OCA (0 μM OCA); ^§^ p < 0.05 vs Gemcitabine alone; ^δ^ p < 0.05 vs 0.5μM OCA + Gemcitabine; ^¤^ p < 0.05 vs Cisplatin alone. b) Apoptosis was evaluated by the Annexin V/PI assay. In mixed iCCA cells, OCA enhanced the apoptotic effect of 10 μM Gemcitabine (72 h of incubation) while no effect was found for Cisplatin. In mucinous iCCA cells, OCA failed to influence the apoptotic effect of Gemcitabine and Cisplatin. Data represent means ± SD of N = 12 independent experiments. * p < 0.05 vs controls without OCA (0 μM OCA); ^§^ p < 0.05 vs Gemcitabine alone.

Gemcitabine and Cisplatin represent the current standard of care for the treatment of CCA [5]. We have previously demonstrated [19] that Gemcitabine and Cisplatin inhibit proliferation and induce apoptosis in mixed and mucinous iCCA primary cell cultures. In the present study we tested concentration of Gemcitabine (10 μM) and Cisplatin (20 μM) previously demonstrated to exert maximum inhibitory effects on cell proliferation in our primary human iCCA cell cultures [19]. In mixed and mucinous iCCA cell cultures, treatment with OCA for 72h potentiated the inhibitory effect of Gemcitabine on cell proliferation irrespective of low (0.5 μM) or high OCA concentration (1.5 μM) (p < 0.05 vs Ctrl and p < 0.05 vs Gemcitabine alone) (Fig 7a). The inhibitory effect of Cisplatin on mixed and mucinous iCCA cell proliferation was also potentiated by OCA but only at the highest concentration tested (1.5 μM) (p < 0.05 vs Cisplatin alone).

In mixed iCCA cells, 72 hrs of treatment with OCA at 0.5 μM enhanced the apoptotic effect of 10 μM Gemcitabine while Cisplatin did not exert any effect (Fig 7b). In mucinous iCCA cells, OCA failed to influence the apoptotic effect of Gemcitabine and Cisplatin (Fig 7b).

### OCA combined with Gemcitabine or Cisplatin abrogates the colony forming capacity of iCCA cells

**Fig 8:**
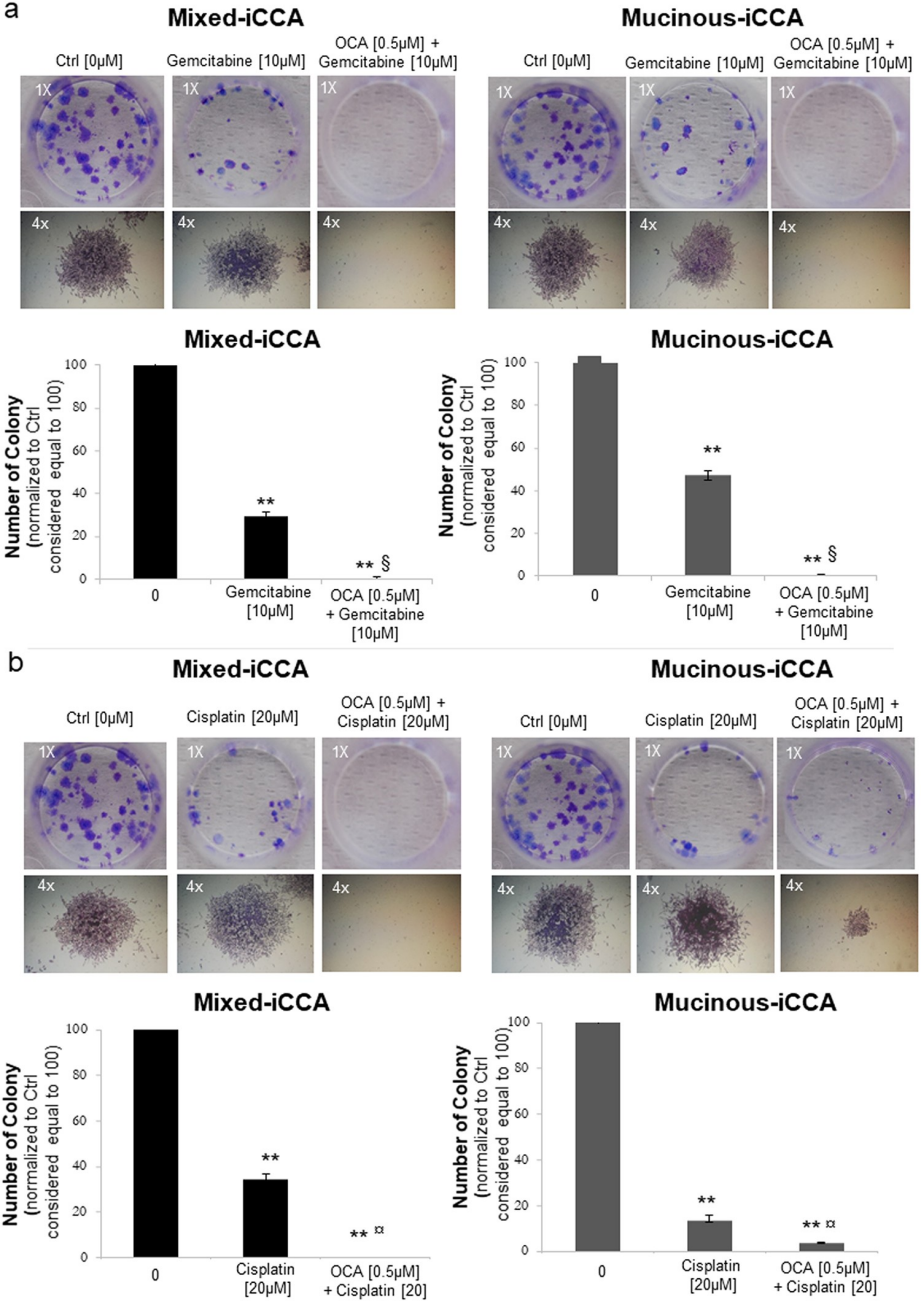
OCA combined with Gemcitabine or Cisplatin abrogates the colony forming capacity of iCCA cells. Colonies formed by mixed and mucinous iCCA cells were fixed with 0.1% crystal violet and counted (a colony was considered a cluster with more than 50 cells). Representative pictures of wells with mixed or mucinous iCCA colonies (magnification 1x) or single colonies (magnification 4x) are shown. The number of colonies was normalised to controls considered equal to 100 (see bar graphs. a) The combination of OCA 0.5 μM + 10 μM Gemcitabine (10 day-treatment) abolished the colony forming capacity of mixed and mucinous iCCA cells. The colony dimension of mucinous and mixed iCCA cells was unchanged by treatment with Gemcitabine alone. b) Cisplatin 20 μM + OCA 0.5 μM completely abolished the colony forming capacity of mixed iCCA cells, while in mucinous iCCA cells colony formation was only decreased compared to Cisplatin alone. The average dimension of colonies was similar between control and Cisplatin alone. Data represent means ± SD of N = 5 independent experiments. ** p < 0.01 vs controls without OCA (0 μM OCA); ^§^ p < 0.05 vs Gemcitabine alone; ^¤^ p < 0.05 vs Cisplatin alone.

The combination of OCA 0.5 μM with 10 μM Gemcitabine (10 days of treatment) abolished the colony formation capacity of mixed and mucinous iCCA cells (p < 0.01 vs untreated controls and p < 0.05 vs Gemcitabine alone), while the treatment with Gemcitabine alone only partially reduced (about 60–70%) the colony formation capacity (p < 0.01 vs controls) (Fig 8a). Furthermore, Gemcitabine alone did not reduce the average dimension of colonies formed by mixed and mucinous iCCA cells, compared to controls. The colony dimension index of untreated mixed iCCA cells was 4.15 ± 0.32 versus 4.34 ± 0.26 in Gemcitabine-treated cells, and the colony dimension index of untreated mucinous iCCA cells was 4.56 ± 0.30 vs 4.70 ± 0.26 in Gemcitabine-treated cells. These differences were not statistically significant.

The combined treatment with 20 μM Cisplatin plus 0.5 μM of OCA completely prevented the colony formation capacity of mixed iCCA cells (10 days of treatment, p < 0.01, Fig 8b) while Cisplatin alone only partially reduced colony formation (p < 0.05 vs untreated controls). In contrast, in mucinous iCCA cells, Cisplatin plus OCA induced only a reduction in number and size of colonies (p < 0.01 vs Cisplatin alone, Fig 8b). Moreover, Cisplatin alone failed to reduce the size of colonies in both mixed and mucinous iCCA cells (colony dimension index of untreated mixed iCCA cells = 4.15 ± 0.32 vs 4.85 ± 0.25 in Cisplatin-treated cells, p = ns; colony dimension index of untreated mucinous iCCA cells = 4.56 ± 0.30 vs 4.40 ± 0.38 in Cisplatin-treated cells, p = ns).

### OCA alone markedly decreases, and combined with Gemcitabine or Cisplatin abrogates, growth of iCCA xenografts

**Fig 9:**
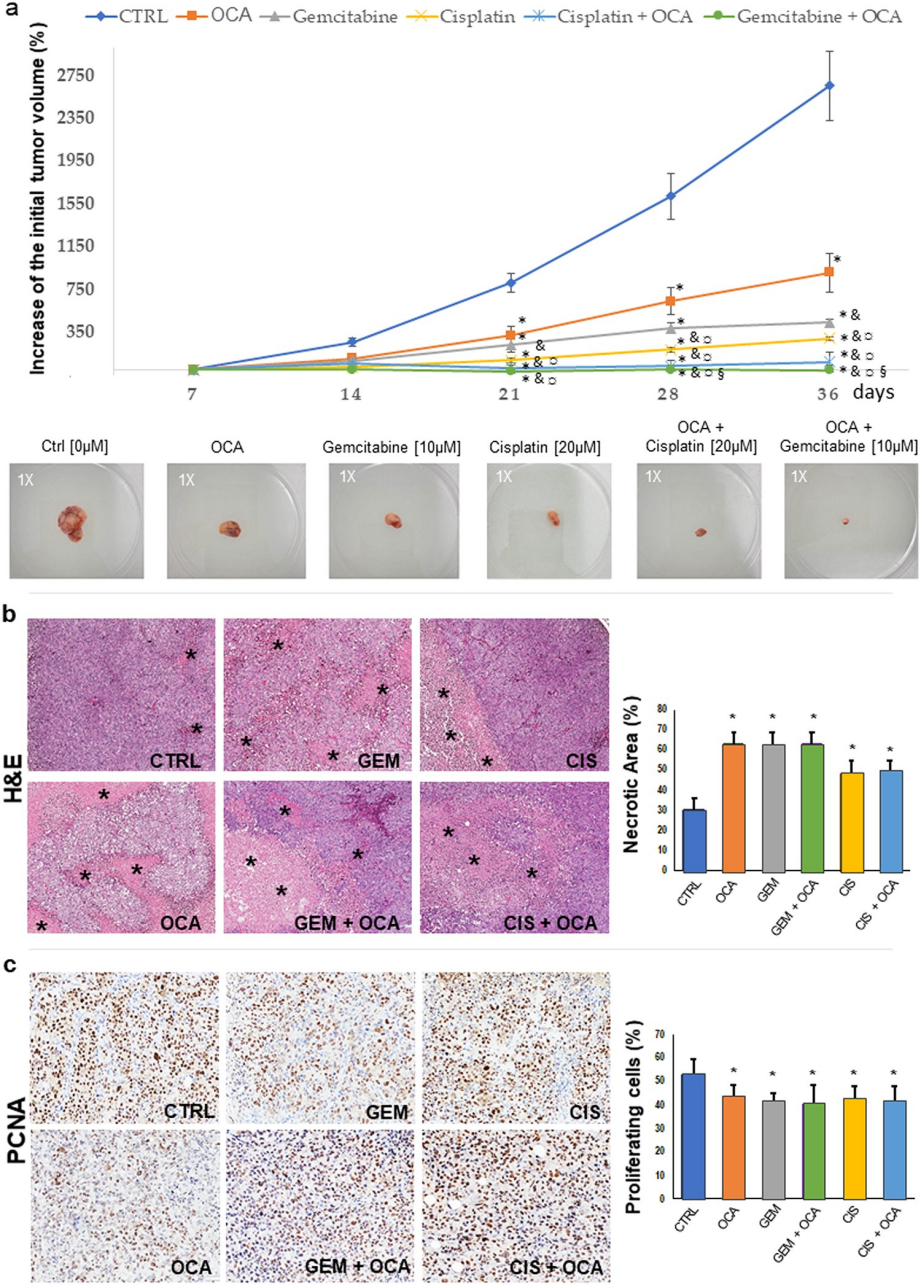
OCA alone markedly decreases and combined with Gemcitabine or Cisplatin abrogates growth of iCCA xenografts. Human mixed iCCA cells from primary cultures were injected (10^6^ cells in 100 μl Matrigel) subcutaneously in male nude BALB/c mice. Tumor xenograft formation was followed by macroscopic inspection. After 4 weeks, when the tumor size was approximately 100 mm^3^ (T_0_) mice were treated with OCA (0.03% w/w, 30 mg/kg) alone or combined with Cisplatin (2.4 mg/Kg in 100 μl) or Gemcitabine (1.05 mg/Kg in 100 μl) for 5 additional weeks. a) Results are expressed as percent increase in tumor volume at different time points. At the end of treatment, tumor volume was markedly lower in mice treated with OCA compared to untreated controls (p < 0.05). Treatment with Gemcitabine or Cisplatin alone decreased tumor volume to a higher (p < 0.05) extent than OCA alone. However, co-treatment of OCA with Gemcitabine or Cisplatin resulted in a tumor volume significantly lower compared to Gemcitabine or Cisplatin alone (p < 0.05). Means ± SE of N = 5 for each group. * p < 0.05 vs controls, ^&^ p < 0.05 vs OCA, ^¤^ p < 0.05 vs Cisplatin alone, ^§^ p < 0.05 vs Gemcitabine alone. Photographs (1x magnification) are representative of tumors formed after 5 weeks of treatment. b) Hematoxylin and eosin (H&E) stain on tumor samples from control mice (CTRL) and mice treated with OCA, Gemcitabine (GEM), Cisplatinum (CIS), and the indicated combinations. Tumor specimens from treated mice display significantly larger necrotic areas (asterisks) compared to CTRL mice. Histograms represent means ± standard deviations. * p < 0.01 vs controls without OCA (0 μM OCA). c) Immunohistochemistry for Proliferating Cell Nuclear Antigen (PCNA) on tumor samples from control mice and mice treated with OCA, GEM, CIS, and the indicated combinations. Tumor specimens from treated mice are characterized by significantly lower percentage of PCNA^+^ (proliferating) cells compared to CTRL mice. Histograms represent means ± standard deviations. * p < 0.05 vs controls without OCA (0 μM OCA).

iCCA cells from primary cultures were injected subcutaneously in the flank of male nude BALB/c mice and tumor formation was followed by macroscopic inspection. After 4 weeks, when the tumor size was approximately 100 mm^3^ (T_0_) mice were treated with OCA (0.03% w/w, 30 mg/kg) alone or combined with Cisplatin (2.4 mg/Kg in 100 μl) or Gemcitabine (1.05 mg/Kg in 100 μl) for 5 additional weeks. Tumor volume (Fig 9a) was significantly lower in mice treated with OCA with respect to untreated controls (p < 0.05). Treatment with Gemcitabine or Cisplatin alone decreased tumor volume to a higher (p < 0.05) extent than OCA, but co-administration of Gemcitabine + OCA or Cisplatin + OCA resulted in a significantly lower tumor volume in comparison with Gemcitabine or Cisplatin alone (p < 0.05) indicating that OCA exerts an additive anti-neoplastic effect when combined with the two chemotherapeutics (Fig 9). No statistically significant differences between Gemcitabine + OCA or Cisplatin + OCA treatments were found.

Histomorphology and immunohistochemistry on tumor samples demonstrated that mice treated with OCA, with or without Gemcitabine or Cisplatin, displayed significantly higher necrotic areas (Fig 9b) compared to control mice (p < 0.05). Higher necrosis was paralleled by a lower percentage of PCNA^+^ cells (Fig 9c).

Thus, this experiment demonstrates that co-administration of OCA with Gemcitabine or Cisplatin almost completely abolishes tumor formation in xenograft models.

## Discussion

The main findings of our study indicate that in human iCCA primary cell cultures: 1) FXR is markedly down-regulated; 2) the potent FXR selective agonist, OCA, upregulated FXR expression and impaired cell proliferation at very low concentrations (i.e. 0.01–0.5 μM); 3) *in vitro*, OCA induced apoptosis of iCCA cells and impaired their colony and spheroid formation as well as invasion and migration capacities; 4) OCA exerted additive anti-proliferative and pro-apoptotic effects with Gemcitabine or Cisplatin on iCCA cells; 5) *in vivo*, OCA impaired the growth of xenografts and exerted additive effects with Gemcitabine or Cisplatin. Taken together, our results indicate that OCA plays anti-neoplastic effects against iCCA.

Belonging to the super-family of metabolic nuclear receptors, FXR is the main bile acid sensor in liver and intestine, regulating the transcription of key genes involved in bile acid synthesis and transport, lipid and glucose metabolism, cell proliferation and inflammatory processes [7–11, 23, 27]. Therefore, given the key role of FXR in controlling major cell functions, it is perhaps not surprising that it is markedly down-regulated in a neoplastic cell that is typically anarchic and unhooked from any form of regulation [13, 14, 28, 29]. Consistently, FXR^−/−^ mice spontaneously develop liver cancer beyond 12 months of age, FXR down-regulation has been described in different cancers and during colon cancer development and progression [28, 30–34].

We compared the expression of FXR in iCCA with a preparation of hBTSCs, since the high representation of CSCs in our primary cultures of both mucinous and mixed iCCA suggests an origin from stem/progenitor cells located in canals of Hering or intrahepatic peribiliary glands [35]. However, in previous studies, the lower expression of FXR in CCA cells was also demonstrated in comparison with normal cholangiocytes or peritumoral non-neoplastic tissues [29]. Indeed, in a large number of CCA patients from two independent cohorts, lower FXR expression was observed in tumors compared to surrounding normal liver tissue and, most importantly, a lower level of expression in poorly-differentiated versus moderate/well differentiated tumors, indicating a direct correlation of FXR expression with CCA aggressiveness and prognosis [29].

In the present study, we used primary iCCA cell cultures that recapitulate the typical features of human CCA, including high representation of CSC markers and EMT traits, and high cancerogenic potential *in vitro* and *in vivo*. *In vitro*, we evaluated the effects of OCA on the classic tests of cancerogenesis, including proliferation, apoptosis, clonogenicity, spheroid formation, migration and invasion. As far as proliferation is concerned, we demonstrated in dose-response curves that OCA exerts a significant anti-proliferative effect at concentrations as low as 0.01–0.05 μM. OCA has been approved for clinical use in patients with the autoimmune disease primary biliary cholangitis at oral doses of 5–10 mg/daily, resulting in a plasma concentration of OCA approximately 0.3 μM [36, 37].

Like endogenous bile acids, OCA undergoes extensive glyco- and tauro-conjugation, with less than 1% of OCA remaining unconjugated in serum [37, 38]. All the *in vitro* effects of OCA documented in the present study on iCCA cells occurred at concentrations compatible with the plasma concentrations reached by OCA in treated patients. We should also consider the marked down regulation of the bile acid transporter ASBT in iCCA cells; the relative hydrophobicity of OCA, however, allows passive entrance into the cell, as demonstrated in previous studies [38, 39].

Most importantly, the anti-proliferative effect of OCA, CDCA and UDCA in iCCA cells corresponds to the profile of the agonistic effect on FXR [40]. OCA is 100-fold more potent than CDCA in FXR activation, while UDCA shows no FXR activation properties [37]. Interestingly, here we have provided evidence of a direct effect of OCA on FXR expression, and on downstream FXR-activated pathways. Indeed, OCA administration resulted in the upregulation of FXR, and activation of downstream gene SHP. In particular, the dose dependent up regulation of SHP and down regulation of Bcl-xL support our conclusion that the anti-proliferative effects of OCA in iCCA cells are mediated by FXR agonism. Furthermore, our results are in agreement with several previous studies demonstrating that OCA effects are mediated by FXR activation both *in vitro*, in hepatocytes from FXR KO mice [41], and *in vivo* [42, 43]. Conversely, data are lacking on the inhibition of CCA growth by OCA via FXR-independent pathways.

OCA not only inhibited proliferation, but it also induced apoptosis and counteracted invasion and migration in our iCCA cell cultures. In general, these effects were more evident in mixed rather than mucinous iCCA, consistent with the higher aggressiveness and the worst prognosis of mucinous iCCA demonstrated in different clinical studies [1, 2, 44–46]. Our data show that OCA induces less apoptosis in mucin compared to mixed iCCA cells and has minimal effects on migration and invasion of mucinous iCCA cells in the wound-healing and invasion assays. This further supports the higher resistance of mucinous iCCA cells to OCA effects but, at the same time, indicates that inhibition of cell migration and invasion observed in mixed iCCA is not just the consequence of impaired proliferation and pro-apoptotic effects, which have been observed also in mucinous iCCA cells.

Accumulation of bile acids has been considered to play a pathogenic role in CCA development, thus strategies aimed at inhibiting their synthesis or promote elimination are under evaluation [11, 47]. Among them, FXR activation plays a central role, by inhibiting bile acid synthesis via Cyp7α1, and by exerting several anti-cancer effects, such as suppression of β-catenin expression and function [24, 48]. Very recently, in patients with iCCA, FXR expression has been found to be negatively correlated with the IL-6 level [49]. It is well known that IL-6 has an integral role in iCCA prognosis by acting as a growth and survival factor for CCA cells. FXR activation inhibited ICC growth and metastasis via IL-6 suppression *in vitro* and *in vivo* where counteracting epithelial-mesenchymal transition is a key mechanism [49]. Thus, compelling evidence is emerging on mechanisms which link FXR activation and effects hindering cholangiocarcinogenesis. We have also evaluated effect of OCA on SHP and Bcl-xL gene expression. SHP is a typical target gene of FXR activation and Bcl-xL is a key gene involved in modulating apoptosis pathways [24]. Our results show that OCA increased the expression levels of SHP gene, and down-regulated Bcl-xL gene expression, confirming the activation of apoptotic pathways [24]. In accordance with these observations, growing evidence demonstrates that SHP has a tumor suppressor function and represents an active component in apoptosis signaling [24]. Therefore, based on the present results and previous observations, we suggest that FXR activation induces SHP gene transcription which in turn induces down regulation of Bcl-xL and therefore apoptosis.

The most clinically relevant result of our study, however, was seen in the *in vivo* iCCA xenografts, since OCA alone induced more than 50% decrease of tumour growth and further exerted additive effects with Gemcitabine and Cisplatin. Strikingly, OCA combined with Gemcitabine or Cisplatin completely inhibited xenograft growth. iCCA xenografts recapitulate the desmoplastic features of the original cancer with high representation, in the neoplastic mass, of mesenchymal vs epithelial components [3]. Interestingly, OCA alone induced areas of necrosis in the neoplastic mass in addition to inhibiting cell proliferation, the latter being consistent with *in vitro* results.

CCA patients are frequently diagnosed at advanced stages, when surgical curative treatments are inapplicable [50, 51]. In addition, very frequently, the presence of cirrhosis or cholestatic liver damage in iCCA patients contraindicates the use of chemotherapeutics [52, 53]. For all these considerations, drugs with a high safety profile should be used but, currently, no drug with this characteristic has been approved. OCA could represent an ideal candidate, since the major side effect is itching, which is however manageable by dose-adjustments [54].

Our data are consistent with previous reports showing the inhibitory effect exerted by GW4064, a synthetic non-steroidal isoxazole-based FXR agonist, on the subcutaneous growth of CCA cells in nude mice [55]. In addition, FXR activation has been shown to promote chemosensitization [24, 25] and this is consistent with our findings showing, *in vitro* and *in vivo*, additive anti-neoplastic effects of OCA administered together with Gemcitabine or Cisplatin. In addition, OCA could be tested as chemopreventive agent in patients at high risk of iCCA such as PSC.

In conclusion, our study demonstrates relevant *in vitro* and *in vivo* anti-cancer effects of OCA against human iCCA primary cultures and this reinforces accumulating evidence of FXR as a therapeutic target in patients with CCA. Given the good safety profile, clinical trials testing OCA alone or in combination with chemotherapeutics in surgically untreatable CCA patients could offer interesting perspectives.

## Abbreviations

BS: bile salts
CDCA: Chenodeoxycholic acid
cDNA: complementary DNA
CSC: Cancer stem cell
DMEM: Dulbecco’s modified Eagle Medium
DMSO: Dimethyl sulfoxide
dNTP: Deoxyribonucleotide triphosphate
DPBS: Dulbecco’s phosphate-buffered saline
DTT: Dithiothreitol
FACS: Fluorescence-Activated Cell Sorter
FBS: Fetal bovine serum
FXR: Farnesoid X receptor
H&E: Hematoxylin and Eosin
hBTSC: Human Biliary Tree Stem Cell
hEGF: human epidermal growth factor
iCCA: intrahepatic cholangiocarcinoma
IF: immunofluorescence
IgG: Immunoglobulin G
miRNA: micro-RNA
M-MLV: Moloney-Murine leukemia virus
OCA: Obeticholic acid
Oligo(dT)_12-18_ Primer: thymidine oligonucleotides
PBG: Peribiliar Gland
PCNA: proliferating cell nuclear antigen
qPCR: quantitative Polimerase Chain Reaction
SD: standard deviation
SE: standard error
UDCA: Ursodeoxycholic acid
